# Effect of Irrigation Activation Techniques on Periapical Organic Tissue Dissolution in Simulated Immature Teeth: An Ex Vivo Study †

**DOI:** 10.3390/bioengineering13010089

**Published:** 2026-01-13

**Authors:** Kadriye Demirkaya, Hulde Korucu, Zeliha Ugur Aydin, Sevgi Bulak Yeliz

**Affiliations:** 1 Department of Endodontics, Gulhane Faculty of Dentistry, University of Health Sciences, Ankara 06010, Turkey; korucu.hulde@gmail.com (H.K.); zlhugur@gmail.com (Z.U.A.); sevgi.bulak9898@gmail.com (S.B.Y.); 2 Department of Endodontics, Henry M. Goldman School of Dental Medicine, Boston University, Boston, MA 02118, USA

**Keywords:** regenerative endodontics, irrigation activation techniques, ultrasonic irrigation (UI), photon-induced photoacoustic streaming (PIPS), shock wave-enhanced emission photoacoustic streaming (SWEEPS), apical tissue dissolution

## Abstract

**Background/Objectives**: Effective removal of organic tissue extruded beyond the apex is crucial in regenerative endodontics, particularly in teeth with immature apices; therefore, this study aims to compare the efficacy of standard needle irrigation (SNI), ultrasonic irrigation (UI), photon-induced photoacoustic streaming (PIPS), and shock wave-enhanced emission photoacoustic streaming (SWEEPS) techniques in dissolving periapical tissue in a simulated model. **Methods**: Sixty single-rooted human premolars and sixty bovine palatal mucosa specimens were used. A custom model was created by placing mucosal tissue in contact with the apical area. Specimens were divided into four groups (n = 15) according to the irrigation method: SNI, UI, PIPS, and SWEEPS. Each canal received 15 mL of 2% NaOCl. Tissue samples were weighed before and after treatment. One-way ANOVA and Tukey’s post hoc test were used for statistical analysis (*p* < 0.05). **Results**: UI showed significantly less tissue dissolution than the other methods (*p* < 0.05). SNI, PIPS, and SWEEPS showed no significant differences (*p* > 0.05). **Conclusions**: All methods led to tissue loss, but UI was significantly less effective. SNI, PIPS, and SWEEPS performed similarly.

## 1. Introduction

Developmental anomalies, dental trauma, or extensive carious lesions may result in pulp necrosis and subsequently interrupt root maturation in immature permanent teeth [1,2]. This clinical scenario presents substantial therapeutic challenges, particularly due to the presence of necrotic pulp tissue, open apices, and structurally compromised dentinal walls [3]. In these cases, conventional mechanical instrumentation is often contraindicated or limited because of the risk of weakening the already fragile root structure; therefore, canal disinfection primarily relies on chemical irrigation and intracanal medicaments [4,5]. Regenerative endodontic procedures in immature teeth require effective removal of organic tissue remnants while minimizing the risk of irrigant extrusion beyond the apical foramen. Irrigation activation systems have been proposed to enhance organic tissue dissolution; however, increased hydrodynamic activity may also elevate the risk of irrigant extrusion, particularly in teeth with wide apical openings. Therefore, evaluating both the efficacy and safety of irrigation activation systems is essential for their clinical applicability in regenerative endodontics. To enhance the efficacy of irrigation, various activation techniques have been proposed [6]. Nevertheless, these approaches may increase the likelihood of apical extrusion of irrigants, particularly in teeth with open apices [7]. The extrusion of sodium hypochlorite or other irrigating agents into the periapical area can result in tissue dissolution and cytotoxic damage to stem cells, ultimately compromising the healing and regenerative potential of the affected tissues. Consequently, there is a pressing need for an ideal irrigation activation method that offers effective disinfection while minimizing adverse outcomes such as apical extrusion and unnecessary tissue damage. The development of such a technique remains a key objective in the endodontic management of immature teeth [8].

Standard needle irrigation (SNI) remains a commonly utilized approach in root canal therapy, primarily due to its ease of use and cost-effectiveness. Despite these advantages, SNI exhibits several critical limitations, including insufficient penetration of irrigants into dentinal tubules and an increased risk of apical extrusion of the irrigation solution. These shortcomings significantly reduce its overall disinfection efficacy and have driven the advancement of more sophisticated irrigation activation methods aimed at improving both the reach and performance of irrigation protocols [9].

Ultrasonic irrigation (UI) is defined as a technique in which an ultrasonic device operating at frequencies between 25 and 30 kHz activates the irrigant using a smooth, non-cutting tip inserted into the prepared root canal system [10,11]. This method has been shown to induce acoustic microstreaming and cavitation phenomena within the irrigating solution [11]. The resulting acoustic microstreaming facilitates the removal of intracanal debris and smear layer while also mechanically disrupting bacterial biofilms. Consequently, planktonic bacteria released from these disrupted biofilms exhibit increased vulnerability to antimicrobial agents [12].

Laser-activated irrigation (LAI) represents an advanced strategy aimed at enhancing the penetration and efficacy of irrigating solutions within the complex anatomy of the root canal system. Among the various laser systems used, Er:YAG lasers (wavelength 2940 nm) are particularly favored due to their high absorption in water and optimal interaction with aqueous solutions. A notable refinement of this approach is Photon-Induced Photoacoustic Streaming (PIPS), a technique that employs ultra-short, low-energy laser pulses to induce intense cavitation and photoacoustic shock waves within the irrigant [11]. The objective of PIPS is to establish a vigorous three-dimensional fluid motion throughout the canal system while maintaining a minimal thermal effect [11]. This approach minimizes the need for extensive canal enlargement and enables effective delivery of irrigants to apical areas, lateral canals, and anatomical complexities such as isthmuses [13]. SWEEPS (shockwave-enhanced emission photoacoustic streaming) represents one of the most advanced laser-assisted irrigation activation methods currently introduced in endodontics. This technique utilizes a dual pulse Er:YAG laser emission, where two sequential pulses are delivered within an optimal temporal interval to induce enhanced cavitation dynamics in the irrigant. The interaction of these pulses generates secondary shockwaves that intensify fluid streaming within the root canal system. Compared with conventional photoacoustic approaches, the dual-pulse modality of SWEEPS produces higher pressure gradients and more pronounced fluid acceleration, thereby optimizing irrigant penetration into anatomically complex regions of the canal system. Owing to its physical mechanism of shockwave reinforcement, SWEEPS has been highlighted in the literature as a significant advancement over single-pulse laser activation systems, particularly for improving irrigant streaming efficiency in narrow and apical areas of the canal [14,15]. Although the effectiveness of laser and other irrigation activation systems has been evaluated in various tooth models, studies that address these systems in models simulating immature teeth with open apices, together with disinfection safety in the context of regenerative endodontics, remain limited. The present study aims to evaluate the use of these systems in teeth with open apices by jointly considering both efficacy and safety aspects.

The aim of this study was to evaluate the effectiveness of different irrigation activation systems in promoting organic tissue dissolution in a simulated immature tooth model, while simultaneously assessing their potential to cause irrigant extrusion beyond the apical area. The null hypothesis asserts that there will be no statistically significant differences in the extent of periapical tissue dissolution among these irrigation activation protocols when applied under standardized experimental conditions. This study was previously presented in part at an international conference [16].

## 2. Materials and Methods

This study was conducted following ethical approval obtained from the University of Health Sciences Gülhane Scientific Research Ethics Committee (Ethics Number: 2024-327). The sample size was calculated based on a power analysis using G*Power software 3.1.2 (Universitat, Düsseldorf, Germany) with an alpha error probability of 0.05 and a power of 80% (effect size = 0.25) concerning a recent study of similar design [17]. The power analysis indicated that a minimum of 15 specimens per group—amounting to a total of 60 samples—was necessary to achieve sufficient statistical power. The experimental model consisted of 60 extracted human mandibular premolar teeth, each with a single root and canal, alongside 60 samples of bovine palatal mucosa. The extracted teeth were selected based on strict inclusion criteria, excluding any with internal or external resorption, coronal caries, restorations, structural defects such as cracks or fractures, or a history of root canal therapy. To confirm compliance with the inclusion criteria, all selected teeth were radiographically evaluated from both buccolingual and mesiodistal projections, ensuring the presence of a single root and canal and the absence of internal or external resorptive defects. The teeth were subsequently immersed in a 5.25% sodium hypochlorite (NaOCl) solution (Cerkamed, Cerkamed Company, Stalowa Wola, Poland) for 48 h to eliminate any residual organic matter adhered to the external surfaces. After this decontamination step, remaining tissue residues were meticulously removed using a periodontal curette under visual inspection. The cleaned specimens were then preserved in a 0.1% thymol solution at room temperature until their allocation to experimental procedures.

Access cavities were created using a high-speed handpiece equipped with a diamond bur (Horico, Berlin, Germany). Apical patency was confirmed using a #10 K-file (Perfect, Shenzhen, China). After confirming apical patency using a #10 K-file (Perfect, Shenzhen, China), the working length was established at 1 mm short of the apical foramen. Root canal instrumentation was carried out with ProTaper Next (Dentsply Maillefer, Ballaigues, Switzerland) rotary files up to the X3 size at the established working length, following the manufacturer’s specified torque and rotational speed settings. After each instrument change, irrigation of the root canals was performed for 30 s using 2 mL of 2% NaOCl (Cerkamed), delivered with a 30G side-vented needle (Ultradent, South Jordan, UT, USA). To ensure standardization across specimens, reference landmarks were established 5 mm coronally and 11 mm apically to the cemento-enamel junction. Tooth structure at these lines was carefully removed using a diamond disc (Bredent, Senden, Germany), resulting in standardized specimen dimensions with crown lengths of 5 ± 1 mm and root lengths of 11 ± 1 mm. In the coronal 5 ± 1 mm portion of each canal, a simulated pulp chamber—serving as the irrigant reservoir—was performed using a #245 carbide fissure bur [18] in order to ensure standardized cutting dimensions and to enhance the reproducibility of the experimental conditions [15]. All cutting procedures were performed under continuous distilled water irrigation using a high-speed handpiece in order to minimize thermal artifacts and to prevent undesirable structural alterations in dental hard tissues. In order to mimic the anatomical characteristics of an immature apex, the standardized apical opening was enlarged to a diameter of 1.5 mm using sequential Gates Glidden drills from sizes #1 to #6 (VDW, Munich, Germany) [19]. The root canals were sequentially irrigated with 5 mL of 2% NaOCl (Cerkamed) for 60 s, followed by 5 mL of 17% EDTA (Cerkamed) for 60 s, and finally rinsed with 5 mL of distilled water for 30 s.

**Figure 1:**
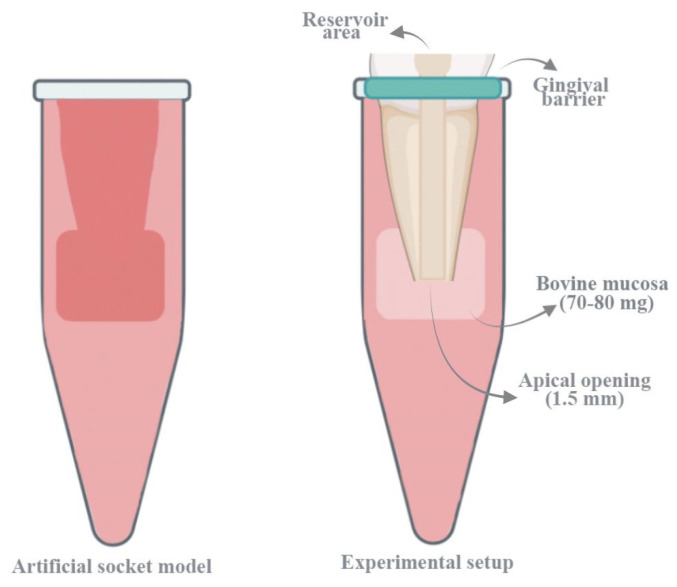
Experimental model.

The experimental model was adapted from the protocol outlined by Ribeiro et al. [17] (Figure 1). Two layers of modeling wax, each 5 mm in diameter and 3 mm in height, were positioned at the apical end of the tooth and conformed to its surface using a heated spatula. To establish an effective seal and prevent resin infiltration, the external root surface was coated with a protective lacquer prior to embedding the specimen in acrylic resin. To minimize thermal deformation of the wax caused by the exothermic polymerization of the resin, the entire assembly was immersed in ice water until complete setting occurred. Once the acrylic block had hardened, a reference mark was made at the junction of the tooth and acrylic surface. The tooth was then removed from the mold, and the wax plugs at the apex were carefully extracted. A second reference mark was placed 2 mm apical to the first, aligned parallel to it.

To simulate periapical tissues, full-thickness sections of bovine palatal mucosa were harvested from slaughterhouse specimens and stored at −18 °C until use. Before testing, the samples were thawed for 30 min in physiological saline at room temperature. The average tissue weight was approximately 70–80 mg. Additionally, in order to evaluate potential changes in mucosal specimen weight that might occur solely due to the processes of assembly and disassembly, independent of irrigation, a control group without irrigation was included in the study. In this group, pre- and post-assembly weight measurements of mucosal specimens were performed using a precision balance with an accuracy of ±0.001 g, and the obtained values were statistically analyzed. The analyses revealed that the assembly and disassembly process did not cause a significant change in mucosal tissue weight (*p* > 0.05) and did not result in measurable tissue loss. This finding indicates that the specimen preparation process preserved the integrity of the mucosal tissues and that the experimental results can be reliably interpreted as independent of the effect of irrigation. Mucosal samples were excluded if the distance between the first reference mark and the acrylic surface was found to be less than 2 mm. Once thawed and gently dried with absorbent paper, each specimen was weighed three times using a high-precision balance, and the mean value was recorded as the initial weight. The mucosal samples were then placed in the simulated periapical compartment of the model, with the epithelial layer facing the acrylic and the connective tissue oriented towards the apical root surface. The tooth was reinserted into the model and subjected to a 25 gf compressive force using a Universal Testing Machine (Instron Corp, Canton, MA, USA) until the first reference line was precisely aligned with the edge of the acrylic block. To maintain uniform back pressure during irrigation, the interface between the acrylic and the cervical portion of the root was sealed using a light-curable gingival barrier material (OpalDam, Ultradent Products, Inc., South Jordan, UT, USA). A #100 endodontic plugger (Dentsply Maillefer, Ballaigues, Switzerland) was then used to gently compact any tissue that had entered the root canal lumen [17].

Each of the 60 bovine mucosal specimens was individually numbered from 1 to 60. Random allocation into experimental groups was carried out using a computer-generated sequence obtained from an online randomization tool (www.random.org). The resulting allocation table distributed the samples into four equal groups (n = 15 per group), corresponding to the irrigation activation techniques under investigation: standard needle irrigation (SNI), ultrasonic irrigation (UI), photon-induced photoacoustic streaming (PIPS), and shock wave emission enhanced photoacoustic streaming (SWEEPS).

**Figure 2:**
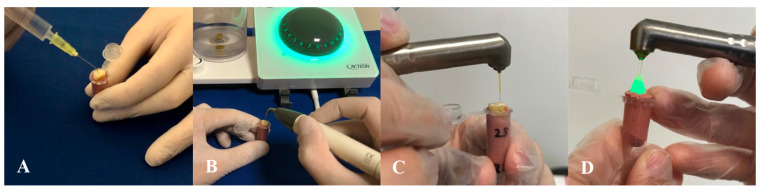
Schematic representations of the irrigation activation techniques ((**A**): standard needle irrigation [SNI], (**B**): ultrasonic irrigation [UI], (**C**): photon-induced photoacoustic streaming [PIPS], and (**D**): shock wave-enhanced emission photoacoustic streaming [SWEEPS]) and their application to extracted human tooth specimens.

SNI: A 30-gauge side-vented needle (Ultradent, South Jordan, UT, USA) was introduced into the root canal at a position 1 mm short of the working length (Figure 2A). Using an in-and-out motion with an amplitude of 3–4 mm, 5 mL of 2% sodium hypochlorite (NaOCl; Cerkamed) was delivered over a 30 s period, completing one cycle of irrigation. This procedure was repeated for two additional cycles, yielding a total of three phases. Final irrigation was carried out with 5 mL of distilled water.

UI: Activation was performed using the Satelec P5 Newtron XS ultrasonic unit (Satelec Acteon, Merignac, France) in conjunction with a #25 Irrisafe tip (Acteon, Merignac, France) (Figure 2B). The device was operated at power level 5. The ultrasonic tip was placed 1 mm short of the working length and activated for 30 s per cycle, for a total of three cycles. During each activation, the tip was allowed to oscillate freely within the canal to maximize acoustic streaming without binding against canal walls.

PIPS: Activation was carried out using a Fotona Light Walker (Fotona, Ljubljana, Slovenia) Er:YAG laser system configured in SSP (Super Short Pulse) mode, with both air and water functions disabled as per manufacturer guidelines. The system was operated at a wavelength of 2940 nm, with pulse energy set at 20 mJ, pulse duration of 25 µs, and a frequency of 20 Hz. The radial laser fiber tip (400/14) was positioned within the irrigation reservoir region. The sequence and volume of irrigation matched those used in the SNI protocol, with three successive 30 s activation cycles (Figure 2C).

SWEEPS: Activation technique was performed using a LightWalker^®^ AT-S Er:YAG laser system (Fotona, Ljubljana, Slovenia). The system was operated at a wavelength of 2940 nm, with pulse energy set at 20 mJ, pulse duration of 25 µs, and a frequency of 20 Hz. The laser fiber tip (600/9) was positioned at the coronal access of the root canal, and activation was carried out according to the manufacturer’s recommendations for endodontic applications (Figure 2D). The sequence and volume of irrigation matched those used in the SNI protocol, with three successive 30 s activation cycles.

In the PIPS and SWEEPS groups, 2% NaOCl (Cerkamed) was continuously replenished in the irrigation reservoir throughout the activation cycles to maintain optimal irrigant levels and efficacy [20]. Upon completion of the irrigation activation procedures, the bovine mucosa specimens were gently retrieved from the experimental setup using forceps. The samples were then carefully dried with absorbent paper and weighed three times using a precision balance. The mean of these three measurements was recorded as the final weight. The extent of tissue dissolution (in milligrams) was calculated by subtracting the final weight from the previously recorded initial weight.

Normality of the data distribution was assessed using the Shapiro–Wilk test. Prior to the statistical analyses, the assumption of homogeneity of variances required for one-way ANOVA was assessed using Levene’s test. After confirming that the variances were homogeneous, intergroup comparisons were performed using one-way ANOVA, followed by Tukey’s post hoc test for multiple comparisons. All statistical analyses were executed using SPSS software (Version 29, IBM Corp., Armonk, NY, USA), with a significance level set at *p* < 0.05.

## 3. Results

Among the irrigation techniques evaluated, UI resulted in the lowest degree of organic tissue dissolution in the periapical area. This finding indicates a more limited interaction between the irrigant and periapical tissues under the hydrodynamic conditions generated by ultrasonic activation. The reduced tissue dissolution observed in the UI group was statistically significant when compared with the other irrigation methods (*p* < 0.05) and was consistent across the specimens within this group.

In contrast, SNI, PIPS, and SWEEPS exhibited comparable levels of periapical tissue dissolution, with no statistically significant differences detected among these groups (*p* > 0.05). These findings suggest that, despite differences in their activation mechanisms, SNI, PIPS, and SWEEPS produced similar extents of organic tissue interaction in the periapical region under the simulated immature tooth conditions. The comparative results for all groups are summarized in Table 1.

## 4. Discussion

The structural fragility of dentinal walls in immature teeth presents a critical limitation to the use of conventional mechanical instrumentation [6,21]. As a result, the disinfection of the root canal system in such cases primarily relies on the effective delivery and performance of irrigating solutions and intracanal medicaments [5]. In an effort to enhance the debridement and antimicrobial efficacy of these agents, a variety of irrigation activation techniques have been proposed in the endodontic literature. However, the characteristically wide apical foramen in immature teeth introduces a heightened risk for irrigant extrusion beyond the apex. Such extrusion events have been associated with adverse clinical outcomes, including pain, burning sensations, and injury to periapical tissues, emphasizing the importance of developing safer irrigation strategies that minimize this risk [22]. Irrigant extrusion in immature teeth occurs under mechanical and hydrodynamic conditions that differ from those in mature teeth. The wide apical opening and the absence of an apical constriction reduce the physiological resistance to apically directed irrigant movement, thereby creating an environment that facilitates the passage of irrigant into the periapical area. Different irrigation methods may influence irrigant extrusion in immature teeth through distinct flow mechanisms. In ultrasonic irrigation, the risk of extrusion is largely associated with the applied hydrostatic pressure and irrigant volume, whereas in ultrasonic and laser-based activation systems, irrigant movement is shaped by energy-mediated flow acceleration and acoustic streaming phenomena. Therefore, comparisons among irrigation methods should be made with consideration of these anatomical and hydrodynamic conditions specific to immature teeth, and extrusion risk should be evaluated for each method within a mechanism-based framework. In the present study, the comparative effects of SNI, UI, and LAI on periapical organic tissue dissolution were investigated using a simulated immature tooth model. The results demonstrated statistically significant differences in tissue dissolution levels among the groups, leading to the rejection of the null hypothesis.

Although the primary focus of this study was the evaluation of organic tissue dissolution, irrigant extrusion was intentionally assessed as a complementary outcome to provide a more clinically relevant interpretation of the findings. In regenerative endodontics, particularly in immature teeth with open apices, enhanced irrigant activation may improve tissue dissolution while simultaneously increasing the risk of apical extrusion. Therefore, the discussion of irrigant extrusion in the present study should be interpreted as an assessment of the safety profile of the irrigation activation systems rather than as an independent research objective.

The concentration, volume, and duration of activation of irrigation solutions are well-documented in the literature as critical factors that can substantially affect the outcomes of endodontic treatment [23]. Accordingly, in the present study, all activation techniques were applied under identical conditions with respect to irrigant concentration, total volume, and activation time to ensure experimental standardization. However, it is recognized that LAI techniques, unlike SNI and UI, may induce outward displacement of irrigant from the reservoir due to energy transfer from the laser. To address this and to remain consistent with protocols reported in previous studies, continuous replenishment of the irrigant was performed throughout the activation period [20].

Ethical limitations associated with the use of human palatal mucosa in experimental research have prompted the exploration of alternative biological models. In this context, bovine palatal mucosa was selected for its histological resemblance to human tissue, coupled with its availability and suitability for standardized laboratory procedures. Although some variation exists in physical and biochemical characteristics, bovine mucosa remains a scientifically acceptable and practically advantageous substitute for modeling periapical soft tissue interactions [24,25]. The findings of this study should be interpreted with caution, as the data were generated under in vitro conditions. Clear methodological description and transparent presentation are necessary to prevent misinterpretation of innovative activation techniques in patients and to allow accurate extrapolation of findings to human clinical practice. The literature indicates that, in experimental studies evaluating the organic tissue dissolving capacity of NaOCl, standardizable soft tissue models are frequently used instead of clinical periapical tissues. Although such tissues do not exactly represent clinical tissues, they provide controlled and reproducible models that allow for the comparative assessment of the relative effectiveness of irrigation systems. Therefore, the mucosal tissue used in the present study should be regarded not as a direct representation of clinical tissues, but as an experimental comparative model.

In the present study, tissue weight loss was used as a comparative experimental parameter to evaluate organic tissue dissolution under different irrigation activation conditions. However, tissue dissolution is influenced not only by the amount of irrigant extrusion but also by factors such as contact time, flow characteristics, and hydrodynamic effects. Therefore, tissue weight loss should be interpreted as an indirect indicator of dissolution behavior rather than as a direct quantitative measure of irrigant extrusion. The absence of direct measurement of extruded irrigant represents a limitation of this study, and the findings should be interpreted within this experimental context.

The present study revealed that none of the tested irrigation activation techniques—SNI, UI, PIPS, or SWEEPS—were able to completely prevent the extrusion of irrigants beyond the apical foramen in simulated immature teeth, as each method led to some degree of periapical tissue dissolution. Moreover, no statistically significant differences were found among SNI, PIPS, and SWEEPS with regard to their capacity for dissolving organic tissue in the periapical area. To date, no comparative study has been identified in the literature specifically evaluating the effects of SNI, UI, PIPS, and SWEEPS on periapical organic tissue dissolution. Therefore, the current findings were interpreted in the context of studies that examined irrigant and debris extrusion in similar models. In contrast to our observations, Azim et al. [26] reported that in single-rooted mature teeth, using an artificial root socket model, PIPS resulted in greater irrigant extrusion than SNI. Similarly, Vatanpour et al. [27] found that in immature molars, SNI caused more extrusion compared to SWEEPS when both methods were applied using the same power output as employed in our protocol. Such variations in outcomes across studies may be explained by differences in experimental design, including variables such as root morphology, preparation dimensions, and the concentration and volume of irrigants used. In contrast to the aforementioned findings, Genç Şen et al. [28] reported no difference in irrigant extrusion between SNI and SWEEPS in single-rooted teeth subjected to both working length and over-instrumentation protocols, aligning with the results of our study. Similarly, Arslan et al. [29], in their use of a modified model involving single-rooted mature mandibular premolars, observed no significant difference between SNI and PIPS even when different power settings (0.3–0.9 W) were applied. The lack of significant differences in periapical tissue dissolution among SNI, PIPS, and SWEEPS observed in our study may be linked to the shared dependence of these techniques on the chemical efficacy of the irrigant and its ability to access target areas. Although each method differs in terms of energy transmission and fluid movement, limitations in irrigant penetration and energy delivery at the periapical level may have minimized any measurable distinctions among these approaches.

In this study, it was observed that UI resulted in less organic tissue dissolution in the periapical area compared to SNI, PIPS, and SWEEPS. Contrary to our findings, İnce Yusufoğlu et al. [30] reported no significant difference in debris extrusion between UI and PIPS in moderately curved molars. Similarly, Avcı et al. [31] found no difference between UI and SNI in terms of the amount of debris extruded apically in mature molars using the Myers and Montgomery model. These discrepancies in results might be attributed to methodological variables such as tooth morphology and experimental setups. In agreement with our findings, Taşdemir et al. [32], using the Myers and Montgomery model on maxillary and mandibular mature incisors with the same NaOCl concentration as our study, reported that UI caused less irrigant extrusion compared to SNI. Additionally, Kashikar et al. [33], using a different NaOCl concentration and volume in mature incisors, observed that UI resulted in less irrigant extrusion than SNI. Mitchell et al. [34] also demonstrated that UI caused less irrigant extrusion than SNI in mature single-rooted teeth, despite using a similar NaOCl concentration but a different volume compared to our study. The significantly lower periapical tissue dissolution observed in the UI group may be attributed to the distinct hydrodynamic and energy transmission characteristics of ultrasonic activation. Although UI is effective in enhancing intracanal debridement through acoustic microstreaming, its energy dissipation is largely confined within the root canal space and may not be efficiently transmitted beyond the apical region, particularly in teeth with simulated immature apices. In contrast, laser-activated irrigation techniques such as PIPS and SWEEPS generate photoacoustic shock waves that induce more pronounced fluid movement and pressure fluctuations, potentially increasing irrigant interaction with periapical tissues. Similarly, syringe needle irrigation may allow more direct apical fluid displacement due to positive pressure delivery. Therefore, the reduced tissue dissolution associated with UI in the present model may reflect its more controlled irrigant dynamics and limited apical energy transfer, rather than reduced overall activation efficacy.

This study has several limitations. First, due to the nature of the simulated immature apex model, essential physiological factors such as periapical vascularity, tissue resistance, and fluid dynamics could not be fully replicated. In addition, the use of bovine palatal mucosa as a surrogate for periapical tissues may present biological and structural differences compared with human periapical tissues, thereby limiting the direct clinical extrapolation of the findings. Within the scope of this study, none of the evaluated irrigation approaches—including SNI, UI, and laser-activated irrigation—was able to completely prevent apical extrusion of the irrigating solutions. This finding highlights the inherent difficulty of controlling irrigants under open-apex conditions and suggests that tissue dissolution and extrusion outcomes may have been influenced by multiple factors, including contact time, flow dynamics, and the volume of irrigant used. These limitations should be considered when interpreting the results and underscore the need for further studies aimed at optimizing irrigation activation strategies to enhance both safety and efficacy in regenerative endodontic procedures.

The high coefficients of variation observed in the UI, PIPS, and SWEEPS groups can be explained by the temporally and spatially variable hydrodynamic flow regimes generated by energy-based irrigation activation systems. In a simulated immature tooth model with an open apex, irrigant flow may lead to differences in irrigant–tissue contact time and contact area among samples. This, in turn, contributes to increased intragroup variability. Therefore, the mean values reflect the comparative behavioral trends of the irrigation systems, and the results should be interpreted within an experimental and comparative framework rather than as absolute quantitative measures.

Therefore, future research should focus on the development of advanced ex vivo models and well-designed in vivo and clinical studies to further investigate irrigation activation techniques under conditions that more closely mimic the physiological periapical environment. Particular emphasis should be placed on evaluating different irrigant volumes, activation durations, and fluid dynamics, as well as their potential impact on periapical tissues over time. Such studies are essential not only to validate the present laboratory findings but also to improve the translational value of experimental data and to support the establishment of evidence-based guidelines for clinical endodontic practice.

## 5. Conclusions

Under standardized ex vivo conditions, all irrigation activation methods resulted in measurable periapical tissue dissolution, with SNI, PIPS, and SWEEPS demonstrating greater dissolution capacity than UI. However, given the ex vivo design and the evaluation of a limited number of activation techniques, these findings should be interpreted cautiously and require further validation through well-designed in vivo studies before clinical extrapolation.

## Figures and Tables

**Table 1:** Mean weight loss and standard deviation of organic tissue loss amount (mg) for each group.

Study Group	N/Group	Mean + Std. Dev.
SNI	15	0.030 ± 0.011 ^a^
UI	15	0.011 ± 0.009 ^b^
PIPS	15	0.016 ± 0.015 ^a^
SWEEPS	15	0.030 ± 0.025 ^a^

SNI, standard needle irrigation; UI, ultrasonic irrigation; PIPS, photon-induced photoacoustic streaming; SWEEPS, shock wave emission enhanced photoacoustic streaming. N: Sample size ± SD: Standard deviation. *p* value: Significance level was determined as *p* < 0.05. Statistical differences between rows are indicated by a and b.

## Data Availability

The data that support the findings of this study are available from the corresponding author upon reasonable request. No publicly archived datasets were generated.

## Abbreviations

The following abbreviations are used in this manuscript:

SNI	Standard Needle Irrigation
UI	Ultrasonic Irrigation
PIPS	Photon-Induced Photoacoustic Streaming
SWEEPS	Shock Wave-Enhanced Emission Photoacoustic Streaming
NaOCl	Sodium Hypochlorite
ANOVA	Analysis of Variance
EDTA	Ethylenediaminetetraacetic Acid
